# Protease nexin 1 induces apoptosis of prostate tumor cells through inhibition of X-chromosome-linked inhibitor of apoptosis protein

**DOI:** 10.18632/oncotarget.2921

**Published:** 2015-02-17

**Authors:** Chad M. McKee, Yunchuan Ding, Jianfeng Zhou, Chunrui Li, Liang Huang, Xiangke Xin, Jing He, Joshua E. Allen, Wafik S. El-Deiry, Yunhong Cao, Ruth J. Muschel, Danmei Xu

**Affiliations:** ^1^ Department of Internal Medicine, Tongji Hospital, Tongji Medical College, Huazhong University of Science and Technology, Hubei, China; ^2^ Gray Institute of Radiation Oncology and Biology, Medical Science Division, University of Oxford, Oxford, United Kingdom; ^3^ Penn State Hershey Cancer Institute, Penn State University, Hershey, PA, USA

**Keywords:** PN1, XIAP, prostate cancer, NF-κB, AKT

## Abstract

Protease nexin 1 (PN1) is an endogenous serine protease inhibitor (SERPIN), expressed at high levels in the prostate, and capable of inhibiting the proliferation of prostate cancer cells. We previously showed that PN1-uPA complexes inhibited Sonic Hedgehog (SHH) signalling through engagement of the LRP receptor. Here, we describe an alternative anti-proliferative mechanism through which PN1 expression leads to apoptosis. In prostate cancer cells, increased expression of PN1 led to substantial reduction of XIAP levels and apoptosis mediated through the uPAR, but not the LRP receptor. The alterations in XIAP were effected in two ways 1) via alteration in the NF-κB pathway, a pathway known to signal XIAP transcription and 2) by promoting XIAP instability. The AKT pathway is known to phosphorylate XIAP at serine 87 leading to protein stability and PN1 expression is shown to interfere with this process. As a result of both mechanisms, programmed cell death is substantially increased. Consistent with these observations, reduced PN1 protein correlated with elevated p65/XIAP expression and with higher Gleason scores in human prostate tissue arrays. Thus, PN1 expression appears to differentially down-regulate distinct oncogenic pathways depending upon the cell surface receptor engaged by its complexes and demonstrates a novel molecular mechanism by which the protein can promote tumor cell apoptosis.

## INTRODUCTION

A distinctive feature of tumor growth is uncontrolled cell proliferation that largely bypasses the process of programmed cell death, or apoptosis [1]. As hyper-proliferative states contribute not only to tumor progression but also to chemo- and radiotherapy resistance [2, 3], selective targeting of pro-survival factors in tumor cells is a possible approach to encourage recovery of the apoptotic apparatus. XIAP (X-chromosome-linked inhibitor of apoptosis protein) is a cellular factor that plays a central role in regulating cell death pathways [4]. Because its overexpression is associated with cancer formation and progression [5–7], drugs antagonistic to XIAP have shown promise as cancer therapeutic agents [8].

XIAP is a member of the inhibitor of apoptosis (IAP) family of proteins that target caspases, the effector molecules of cell death [9]. XIAP is able to effectively bind and neutralise the activity of caspase-3, 7, and 9, which together can drive the mitochondrial and death receptor pathways of apoptosis [10, 11]. XIAP is one of eight individual members of the IAP family, but is the most potent inhibitor as determined by strength of binding to its caspase targets [9]. High levels of XIAP have been associated with malignancy, and have also been found to correlate with reduced survival rate and poorer outcomes in clinical settings [12–15]. Consequently, many small molecules or antisense nucleotides have been designed to block XIAP and engage the cell death program [5, 16–18], with varying success. Here, we show evidence that an endogenous serine protease inhibitor, protease nexin-1 (PN1), leads to reduced expression of XIAP, resulting in induction of apoptosis in prostate cancer.

PN1, known alternatively as SerpinE2 [19], is secreted by a variety of cells, including endothelial cells, fibroblasts, macrophages, astrocytes, and cancer cells [20, 21]. PN1 can potently and irreversibly inhibit a variety of serine proteases including urokinase plasminogen activator (uPA), tissue-type plasminogen activator (t-PA), thrombin and plasmin [22]. We have previously described a novel regulatory pathway in which PN1 blocks the invasion of prostate metastatic cells through the regulation of uPA activity [23] and also demonstrated that PN1 influences sonic hedgehog (SHH) levels, providing a pathway by which PN1 can affect tumor cell proliferation [24]. Engagement of the low density lipoprotein receptor-related protein-1 (LRP-1) receptor proved critical for PN1-mediated inhibition of the hedgehog pathway, blocking proliferation in several prostate and pancreas cell lines [24]. Intriguingly, overexpression of PN1 appeared to concomitantly induce an apoptotic program independent of Hedgehog pathway inhibition. Here we explore the mechanism responsible for the effect of PN1 on apoptosis.

We have found that PN1-mediated inhibition of uPA and its signalling through the uPA receptor (uPAR), but not LRP-1, results in a downstream cascade of events leading to a cellular reduction in XIAP. This can occur either *via* 1) an alteration in NF-κB signalling, lessening *xiap* transcription or 2) through a blockade of AKT signalling, preventing the stabilizing phosphorylation of XIAP at serine 87, therefore promoting the protein to degradation. Thus, the PN1-uPA regulatory axis may be capable of triggering apoptosis by modulating survival pathways and as a result the growth of prostate cancer cells.

## RESULTS

### PN1 expression induces apoptosis and decreases XIAP protein levels

We show here and previously [24] that expression of PN1 leads to the decreased growth and increased apoptosis of prostate metastatic cells. Cell death, as determined by TUNEL and Parp cleavage, increased in PC3 cells after PN1 overexpression (Sup. 1A & B). If injected within Matrigel, PC3 cells can be reliably grown subcutaneously as murine xenografts. Previously, we showed that addition of PN1 to Matrigel delayed the growth of these xenografts [24]. Here, increased cell death also occurs in xenografts formed after innoculation with recombinant PN1 in the Matrigel compared to controls (Sup. 1C).

**Figure 1:**
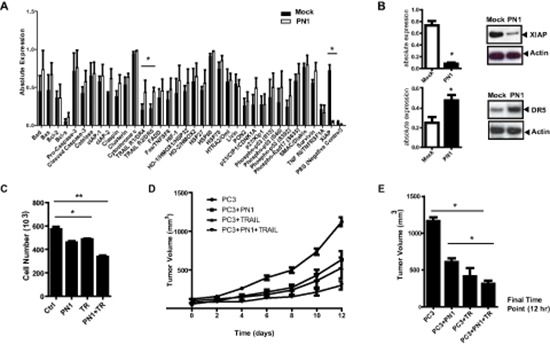
PN1 expression induces apoptosis and decreases XIAP protein levels **(A)** Lysates (300 μg) of PC3 cells (1 × 10^6^) transfected with 2 μg mock (black) or PN1 expressing vector (white) were incubated on an array of 35 pro- and anti-apoptotic proteins. Absolute expression levels were calculated and plotted (*N* = 3, one-way ANOVA, *P < 0.05). **(B)** XIAP and DR5 protein levels validated using immunoblotting and relative intensities measured. (*N* = 3, *t*-test, *P < 0.05). **(C)** Recombinant PN1 (2 μM) or TRAIL protein (200 ng/ml) alone or in combination was added to the medium of PC3 cells (1 × 10^5^) for 24 hrs followed by an overall cell count (*N* = 3,one-way ANOVA, *P < 0.05, **P < 0.01) **(D)** PC3 xenograft tumor volumes from groups pre-treated with PN1 (10 μM) or treated with daily IP of TRAIL protein (40 mg/kg), alone or in combination with PN1 pre-treatment, were measured. (*N* = 5, one-way ANOVA, *P* < 0.05). **(E)** Graphical representation of treatment effects at the 12 day time point (*N* = 5, one-way ANOVA, *P < 0.05).

Having validated that increased levels of PN1 can enhance apoptosis, we sought to determine its effect on known cell death regulatory proteins. Lysates from PC3 cells transfected with an empty vector (Mock) or a PN1 expression vector were evaluated using arrays to detect 35 pro- and anti-apoptotic factors (Figure 1A and Sup. 1D). Of the proteins screened, only XIAP was significantly reduced after PN1 expression. Conversely, levels of death receptors 4/5 (DR4 and DR5) were increased. The changes in XIAP and DR5 levels were verified using western blotting (Figure 1B).

### Combined PN1 and TRAIL treatment induces growth lag in prostate cancer xenografts

These data (Figure 1 and Sup. 1) suggested that PN1 might be an effective pro-death factor, particularly if combined with other agents known to induce apoptosis in cancer cells. DR4/DR5 are receptors for TRAIL (TNF-related apoptosis-inducing ligand), a cellular protein that has shown promise as a cancer cell-selective agent [25, 26]. In PC3 cells, PN1 (2 μM) or human recombinant TRAIL (200 ng/mL) added individually repressed cell numbers after 24 hours in culture. Combination of the two treatments had an additive effect, reducing cell number by roughly 40% (Figure 1C).

To validate these results, PC3 xenografts were generated in SCID mice to test the effect of PN1 on tumor growth. Tumors were formed from cells injected either in Matrigel alone or Matrigel supplemented with PN1 (10 μM). These tumors were then treated with daily administration of TRAIL (40 mg/kg) intraperitoneally after tumors reached 100 mm^3^. The combinatorial effect of PN1 exposure and TRAIL (post-treatment) of PC3 xenografts resulted in slower growth compared to control (1,200 mm^3^ to 300 mm^3^) (Figure 1D & 1E). These data support the hypothesis that PN1 could be a potentially useful adjuvant therapy to treat prostate tumors *in vivo*.

### *XIAP* mRNA expression is reduced by PN1 exposure

**Figure 2:**
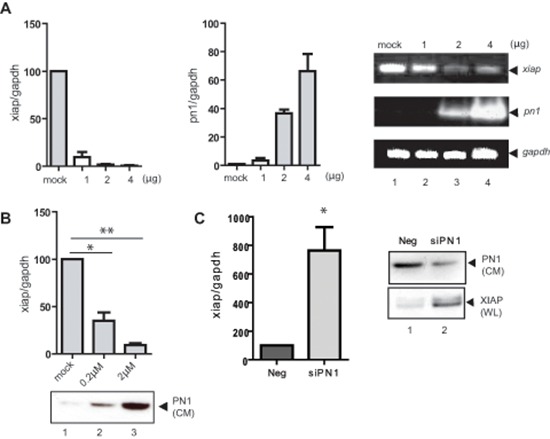
XIAP mRNA expression is reduced by PN1 exposure **(A)** PC3 cells (1 × 10^5^) transfected with 2 μg control vector or increasing PN1 expression vector for 24 h and measurement of *xiap* or *pn1* mRNA levels using qRT-PCR. Right: Products resolved by 1.5% agarose gel. **(B)** PN1 recombinant protein added to conditioned medium of PC3 cells and measurement of *xiap* mRNA transcripts (*N* = 3, one-way ANOVA; *P < 0.05; **P < 0.01). Below: Immunoblotting of cell conditioned medium (CM) for PN1 protein levels. **(C)** PC3 cells (2 × 10^5^) were treated with 10nM negative control siRNA (Neg) or siRNA PN1 (siPN1) for 48 h and measurement *xiap* mRNA transcripts. Right: Immunoblotting of XIAP and PN1 protein levels in whole cell lysates (WL) and cell conditioned medium (CM).

*xiap* RNA levels were determined using qRT-PCR at 24 h following transfection of increasing concentrations of the PN1 expression vector (Figure 2A) or after the exogenous addition of recombinant PN1 protein (0.2 μM, 2 μM) to serum free cell medium (Figure 2B). In both experiments, *xiap* RNA levels were inversely proportional to PN1 levels. Conversely, siRNA against PN1 enhanced *xiap* mRNA amounts (Figure 2C). To evaluate whether inhibition of *xiap* by PN1 is more generalizable, additional cell lines including two human leukemic cell lines (HL-60 and Jurkat) and a cervical cancer cell line (SIHA) were tested. In all three cell lines, PN1 expression reduced *xiap* mRNA levels (Sup. 2).

To determine if PN1-mediated change in XIAP levels was prevalent *in vivo*, brain, liver, lung, bladder, seminal vesicle (Sup. 3A) and prostate (Sup. 3B) tissue lysates from C57WT or PN1 knock mice were obtained. ELISA detection of XIAP showed increases in the brain and the urologic organs (prostate, seminal vesicle, and bladder) when PN1 is genetically ablated from the animal. Interestingly, in lung and liver the trend was reversed. A western blot was performed to further validate XIAP levels and showed substantial increases in the prostates of wild type mice versus PN1 knock-out mice (Sup. 3C).

### PN1 can regulate XIAP by impeding uPA

**Figure 3:**
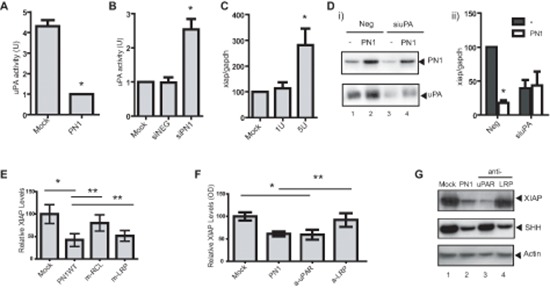
PN1-mediated XIAP regulation requires uPA and uPAR signalling **(A)** In PC3 (1 × 10^5^) cells, uPA activity is reduced following transfection with a PN1 expression vector (2 μg) (*N* = 4, *t*-test, *P < 0.01). **(B)** Knock-down of PN1 *via* (10nM) siRNA increases uPA activity (*N* = 4, one-way ANOVA, *P < 0.01). Mock treatment and scrambled siRNA (NEG) used as controls. **(C)** 1U or 5U of recombinant uPA proteins were added to the medium of PC3 cells (1 × 10^5^) for 24 h and *xiap* mRNA transcripts were measured (*N* = 4, one-way ANOVA, *P < 0.05). **(D)** Immunoblotting of proteins from PC3 conditioned media treated with 10nM control siRNA (NEG) or siRNA uPA (siuPA) and with or without transfection of PN1 (i) and quantitation of *xiap* transcripts (ii). (Two-way ANOVA; *N* = 3, *P < 0.01). **(E)** PC3 cells transfected with control or expression vectors (2 μg) for WT-PN1 or PN1-LRP binding mutant (mLRP) or RCL binding mutant (mRCL), and measurement of *xiap* expression by ELISA (*N* = 4, one-way ANOVA, *P < 0.01). **(F)** PC3 cells transfected with control or expression vectors (2 μg) for WT-PN1 or treated with anti-LRP (50 μg/ml), anti-uPAR (50 μg/ml) blocking antibody for 24 h, and measurement of *xiap* expression by ELISA (*N* = 4, one-way ANOVA with Tukey Test, *P < 0.01; ** refer to similarly significant comparisons between specific groups as denoted by the horizontal lines over the bar graph). **(G)** Immunoblotting of PC3 lysates transfected with PN1 vector or treated with anti-LRP or anti-uPAR blocking antibody for 24 h.

uPA activity has been reported to regulate XIAP transcription [27]. PN1 inhibits the activity of uPA shown here in PC3 cells using overexpression of PN1 vector, or deletion of PN1 with siRNA (Figure 3A–3B). Addition of recombinant uPA (1 or 5 units) to the medium of PC3 cells led to an elevation of *xiap* RNA (Figure 3C). In contrast, down-regulation of endogenous uPA resulted in lowered *xiap* expression (Figure 3D), confirming positive regulation of *xiap* by uPA in this system. When uPA was depleted by siRNA, overexpression of PN1 did not further inhibit *xiap* (Figure 3Dii). Thus, uPA appears essential for the PN1-mediated down-regulation of *xiap*.

### PN1-mediated XIAP regulation requires uPAR signalling

Both LRP-1 and uPAR have been described as membrane receptors that propagate cellular signals after binding protease-PN1 complexes. We previously engineered point mutations into PN1 at its LRP and RCL binding sites [24], both of which are critical for the efficacy of the serpin against Hedgehog signalling. As expected, the RCL mutant (m-RCL) reversed the ability of PN1 to inhibit XIAP. The RCL site is responsible for PN1 binding to its targets, including uPA. However, overexpression of PN1 containing the LRP mutant (m-LRP) did not rescue XIAP at a protein level (Figure 3E). As a result, we then asked whether down-regulation of either of these receptors would alter the extent of *xiap* mRNA. Experiments using siRNA against LRP (Sup. 4, left) failed to show induced alterations of *xiap*. On the other hand, knock-down of uPAR expression by siRNA reduced *xiap* levels (Sup. 4, right).

An additional experiment was conducted at the protein level employing LRP and uPAR blocking antibodies (50 μg/ml each) in conjunction with a sandwich ELISA system for the detection of XIAP. Once more, inhibition of uPAR made a substantial impact on total XIAP protein while its levels were relatively static following LRP blockade (Figure 3F). We also used immunoblotting to further investigate the consequence of blocking membrane receptors on down-stream signalling (Figure 3G). Antibody blockade of LRP-1 altered SHH levels as we found previously [24]. In contrast, XIAP was not significantly affected by blocking antibodies to LRP-1. However, when uPAR was inhibited, XIAP, but not SHH, was down-regulated. This suggests PN1 complexes interact with uPAR but not LRP in mediating XIAP levels and survival signalling. Thus, the response of the cell to PN1 protease complexes is determined by the cellular receptors transmitting the signal.

### PN1 influences NF-κB-mediated regulation of XIAP in prostate tumor cells

**Figure 4:**
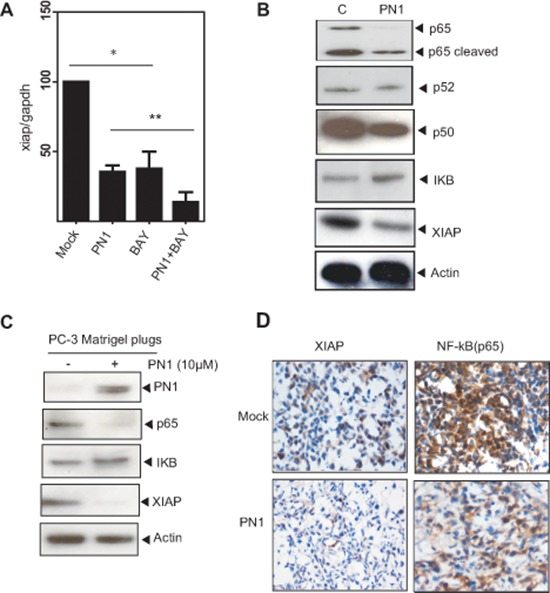
PN1 influences NF-κB-mediated regulation of XIAP in prostate tumor cells **(A)** PC3 cells (2 × 10^5^) transfected with mock or PN1-expressing vector or treated with an NF-κB inhibitor (BAY11-7085, 5 μM), or a combination of both, and measurement of *xiap* mRNA (*N* = 4, one-way ANOVA, *P < 0.05). **(B)** Blotting of NF-κB pathway members from PC3 cells following PN1 overexpression or **(C)** PC3 xenografts +/− pre-treatment with PN1 (10 μM) recombinant protein. **(D)** PC3 xenografts or xenografts pre-treated with PN1 (10 μM), DAB-stained (brown) for p65 and XIAP. Blue stain represents hemotoxylin nuclear staining.

XIAP is transcriptionally regulated by NF-κB signalling [28, 29]. To determine whether PN1 inhibits *xiap* mRNA through the NF-κB pathway, we used an NF-κB inhibitor (BAY 11–7082). As expected, the inhibitor alone reduced *xiap* expression. That effect was amplified by expression of PN1 protein (Figure 4A). Using immunoblotting of cells and murine prostate tissue, we found that alterations in PN1 levels resulted in alterations in expression of various components of the NF-κB pathway. In PC3 cells, elevation of PN1 reduced levels of the NF-κB activating sub-unit, p65 (Figure 4B) and correlated with reduced amounts of XIAP. Interestingly, p65 was not only decreased but cleaved, which typically occurs when there is increased caspase activity [30]. This cleavage can inactivate p65 transcriptional capabilities [30, 31] and supports the hypothesis that reduction in XIAP *via* PN1 leads to a program of cell death.

We further evaluated the expression pattern of these molecules in the Matrigel plug model of tumor growth (Figure 4C–4D). XIAP and p65 levels were reduced in the Matrigel plugs with added PN1. These results are in agreement with the increased apoptosis measured by TUNEL assay (Sup. 1A–1C). Thus, PN1 appears to be important both *in vitro* and *in vivo* in the regulation of XIAP and resulting cell death.

### PN1 destabilizes XIAP through AKT signalling in prostate cancer cells

**Figure 5:**
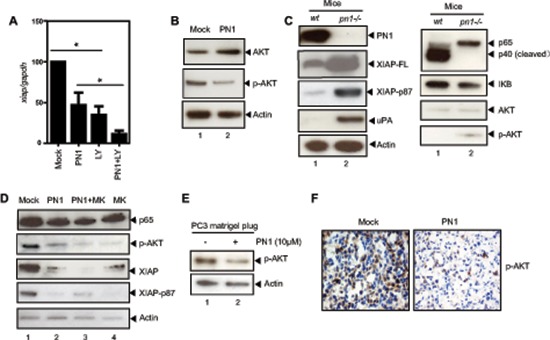
PN1 mediates XIAP stability in prostate cancer cells **(A)** PC3 cells (1 × 10^5^) transfected with 2 μg of Mock or PN1 expressing vector, a PI-3k/AKT inhibitor (LY 294002, 10 μM), or a combination of both, and measurement of *xiap* mRNA (*N* = 4, one-way ANOVA, *P < 0.05). **(B)** PC3 cells transfected with 2 μg Mock or PN1-expressing vectors for 24 h and blotted with indicated antibodies or **(C)** tissue lysates from wild type or *pn1−/−* mice blotted for AKT, XIAP, or XIAP-phospho-serine 87 antibodies. **(D)** PC3 cells transfected with 2 μg Mock or PN1 vector, treated with AKT inhibitor MK-2206 (10 ng/ml) alone, or a combination of both, and measurement of XIAP phosphorylation. **(E)** PC3 xenografts +/− PN1 (10 μM) were blotted *via* immunoblotting as well as DAB-stained (brown) for phospho-AKT **(F)** Blue stain represents hemotoxylin nuclear staining.

Another pathway positively associated with XIAP levels in prostate cancer is AKT [32]. AKT inhibition *via* LY 294002 (10 μM) alone or in combination with PN1 expression, reduced *xiap* expression (Figure 5A). PN1 overexpression resulted in both increased total AKT amounts and decreased phosphorylation (Figure 5B). Previously, Dan *et al* described a system in ovarian cancer cells in which XIAP is phosphorylated by activated AKT at serine-87, an event that acts to stabilize the protein and prevent its auto-ubiquitination [33]. Increased serine-87 phosphorylation and total XIAP were observed in the prostates of *pn1−/−* mice compared to wild type (Figure 5C). Additionally, phospho-AKT was lower in wild type mice than *pn1−/−* mice. These observations suggest that XIAP stability may be preserved in the absence of PN1. This effect can be reversed in PC3 cells by exposure to the AKT inhibitor, MK-2206 (Figure 5D). When treated with 10 ng/mL of MK-2206 following PN1 overexpression, both total XIAP and XIAP phospho-serine-87 were substantially decreased. These data strongly suggest that the AKT pathway is important for XIAP protein stability in prostate cancer cells and that PN1-driven signalling is a factor. The lack of change in p65 protein after MK-2206 treatment shows that AKT signalling is independent from NF-κB in prostate cells. Finally, in Matrigel xenografts, p-AKT levels were lower in Matrigel tumor plugs containing PN1 (Figure 5E–5F). These findings are consistent with PN1 having the capacity to diminish AKT signalling in prostate tumors.

### Elevated p65 and XIAP levels are indicators of advanced prostate cancer

**Figure 6:**
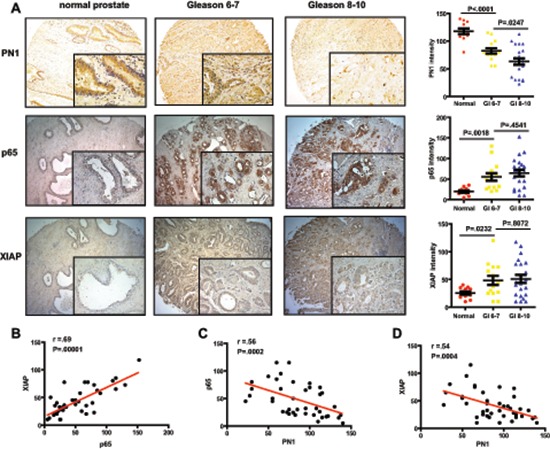
Elevated p65 and XIAP levels are indicators of advanced prostate cancer **(A)** DAB-staining of PN1, p65 and XIAP in human prostatic tissue. Measurement of staining intensity comparing normal prostate tissue (*n* = 12) versus a subdivision of prostate cancer into Gleason scores 6–7 (*n* = 14) or 8–10 (*n* = 22). One-way ANOVA performed. Correlation between groups **(B–D)** calculated using Spearman's *r*-value.

Elevated XIAP has been observed in prostate cancer [14]. Here we show co-expression of p65 and XIAP in the more advanced prostate cancers (Gleason 8–10), and that PN1 protein is concurrently decreased (Figure 6A). XIAP and p65 proteins localize largely to the epithelium, and are sparse in the tumor stroma. The expression of both increased with Gleason score, and exhibits a strong correlation (Spearman correlation analysis, *r* = .69, *p* = .00001). PN1 expression displayed a trend of decreasing in the presence of increasing p65 or XIAP (Figure 6B–6D). Overall, prostate tumors featured high variability in the intensity of all proteins stained, whereas benign control prostate displayed a much tighter range. This could be a reflection of prostate cancer heterogeneity, which is common in the stromal microenvironment, and is also where PN1 protein is primarily expressed [34, 35]. However, the general trend of expression in these human tissues was consistent with a gradual reduction in PN1 concomitant with higher p65/XIAP, matching observed expression patterns in our tissue culture and animal models.

## DISCUSSION

Our previous work demonstrated that PN1 expression in cultured PC3 cells induces cell rounding and anoikis, slows cell division, and promotes cleavage and activation of capase-3 and PARP, all consistent with apoptosis [23, 24]. We have also shown that PN1 can modulate signalling through uPA and the Hedgehog (Hh) signalling pathway, leading to reduced proliferation and invasion of prostate cancer cells, and tumor growth delay in various *in vivo* models [24, 36]. However, overexpression of PN1 greatly increased cell death beyond that caused by inhibition of the hedgehog pathway, suggesting the contribution of the serpin to apoptotic signalling. Here, we have shown evidence that PN1 expression substantially represses XIAP levels. Further investigation also indicates that PN1 could regulate XIAP in two distinct ways, firstly *via* control of RNA levels (Figure 3–4) and also through stabilization of the XIAP molecule itself (Figure 5).

XIAP is the most potent cellular inhibitor of caspases, and an important survival factor. Numerous studies have focused on this protein as a target for degradation in cells that exhibit cancer-like characteristics [37]. XIAP can be inhibited at the physiological level by smac/diablo, which acts to displace XIAP from its target caspases [38]. To date, most pharmacological approaches have concentrated primarily on producing small-molecule inhibitors that mimic this mechanism. We show here that another endogenous molecule, PN1, can inhibit XIAP, in tissue culture and *in vivo*, to promote cell death in prostate tumor cells.

Firstly, PN1 was shown to regulate XIAP at the mRNA level (Figure 2). How PN1 might propagate its signal from the extracellular matrix (ECM) was examined. We found that uPA activity, an ECM target of PN1, correlated with XIAP mRNA levels. This is in accordance with a previous study that implicates uPA in the transcriptional regulation of XIAP in endothelial cells [27]. In that model, uPA regulated transcriptional signalling through binding to its receptor, uPAR. We observed similar results in our study, where biochemical inhibition of uPA or its membrane receptors modulated *xiap* mRNA levels (Figure 3). PN1 is also known to target thrombin, especially in the process of coagulation. However, the effect of PN1 upon thrombin is not widely known to induce apoptosis or proliferation changes in prostate cancer cells. Here the data suggest that complexation of uPA-uPAR in the ECM is itself important to the downstream signalling that drives XIAP. As an uPA inhibitor highly prevalent in the ECM, PN1 is in a position to impede this process and therefore mitigate XIAP expression and ultimately apoptosis in cancer cells.

We showed previously that PN1-uPA complexes preferentially signal through the LRP-1 to control SHH signalling in prostate cancer. In line with these results, blockade of LRP-1 inhibited SHH levels (Figure 3G). However, the same treatment induced no detectable effect on XIAP levels. Instead, down-regulation or blockade of uPAR prevented the cell's ability to express XIAP. The response was similar to that seen after PN1 over-expression. These results suggest that binding to different receptors by PN1-uPA complexes may control different signalling pathways in prostate cancer cells. An intriguing question for future studies is whether uPAR-beta-integrin signalling may contribute to this discrepancy, especially as association of these factors have been linked to cancer progression [39] and may influence invasion of prostate metastatic cells [40].

PN1 expression was shown to alter the NF-κB pathway. NF-κB signalling transcriptionally regulates a number of anti-apoptotic genes, most importantly XIAP itself. In a cell culture model and in matrigel plug tumors (Figure 4), we show that certain NF-κB sub-units, particularly p65/p50, were strongly repressed, consistent with the blockade of the canonical NF-κB pathway. Downstream XIAP levels were reduced in line with decreases in p65 levels. Interestingly, the p65 protein is itself cleaved to a 40 kD fragment when PN1 is highly expressed. This is a process that occurs when p65 is degraded through caspase activation [41], which is consistent with the higher cell death generally observed during increased PN1 expression.

**Figure 7:**
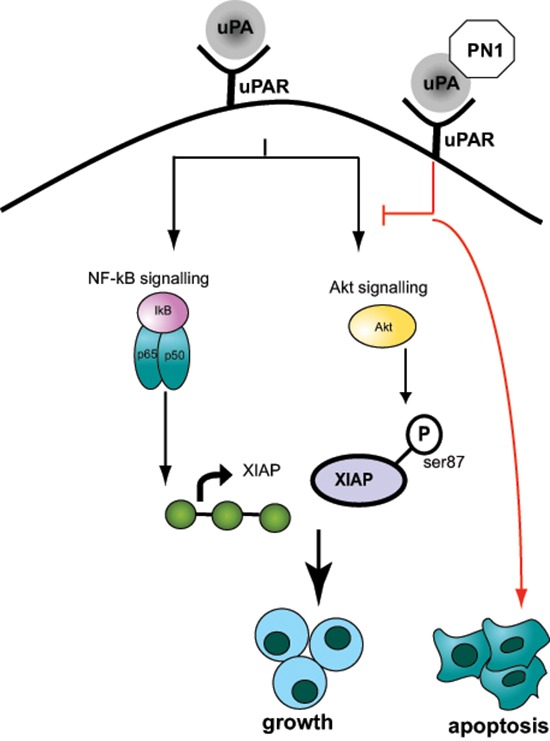
Model Figure PN1 expression mediates apoptosis via regulation of XIAP. PN1 can affect this regulation through down-regulation of the NF-κB pathway activator p65, impeding XIAP transcription. Alternately, PN1 activity can reduce the stability of XIAP by preventing a stabilizing phosphorylation at serine-87, mediated by the AKT pathway. The dual regulation of XIAP is facilitated through inhibition of uPA and altered signalling through uPAR.

A further novel observation made in this study is that the phosphorylation of AKT is altered in mice with genetic ablation of PN1, or in xenografts that have been exposed to PN1 pre-treatments (Figure 5). uPA is able to promote AKT signalling through uPAR [42, 43]. Additionally, prior studies have determined that AKT signalling is associated with the stabilization of XIAP *via* phosphorylation at serine 87 [33]. Our data shows that when PN1 is removed from the system, such as in *pn1−/−* mice, increased total and phosphorylated XIAP is observed. This would suggest protection from auto-ubiquitnation and resistance to cell death. Thus, impeded AKT signalling would represent a second arm of PN1-mediated control of XIAP and help explain why such robust changes in cell death can be observed after expression (Figure 7). Importantly, obstructing the AKT system using specific inhibitors such as MK-2206 does not appear to impact the NF-κB pathway, as p65 levels remain stable. This implies independence of these two signalling pathways (Figure 5D).

These findings reveal novel regulatory targets for PN1 and suggest a greater potential in impeding tumor growth and metastasis. Reducing XIAP with siRNA affects viability in several cell lines and can dramatically sensitize tumor cell to TRAIL mediated apoptosis [44]. These observations match our apoptotic array data showing PN1-induced TRAIL-R1 (DR4) and TRAIL-R2 (DR5) expression simultaneous with decreased XIAP. Treatments of PC3 cells with a combination of PN1 and TRAIL recombinant proteins significantly reduced tumor growth in xenografts (Figure 1D–1E), suggesting a potent sensitization of prostate tumor cells to death signals.

Additionally, staining from human prostate tissue microarrays (TMAs) show an inverse correlation between PN1 expression and XIAP (Figure 6). PN1 levels are decreased in a step-wise fashion from their highest expression in normal prostate tissue to near ablation in the most advanced cancer (Gleason 8–10). Furthermore, advanced prostate cancer expressed significant levels of p65 concomitant with XIAP. This data could suggest that another avenue of treating severe CaP may entail blocking both NF-κB signalling in addition to XIAP.

Our data provides evidence that PN1 can regulate signalling by neutralising complexes uPA-uPAR and altering downstream pathways that contribute to the transcriptional activation and stabilization of the survival factor XIAP in prostate cancer cells. As a result, PN1 levels may affect the overall severity of prostate cancer. Therefore, induction of PN1-mediated signalling may be useful for enhancing XIAP-specific inhibition by small molecule compounds in cancer therapy.

## METHODS

### Animals

pn1−/− mice were a generous gift from MC Bouton (Université Paris, Paris, France). All knock out animals were compared against littermate controls. All animal experiments and protocols were reviewed and performed in accordance with UK Home Office and Oxford University regulations.

### Plasmids and mutagenesis

pcDNA3-PN1 was a kind gift from Dr Peter Andreasen's lab (Aarhus, Denmark). A range of point mutations were generated previously as described [24].

### Cell culture and treatment

Cancer cell lines PC3, SIHA, Jurkat and HL-60 cells were obtained from the American Type Culture Collection (ATCC) and regularly tested to ensure the absence of Mycoplasma contamination (MycoAlert, Lonza). New stock vials were thawed every 3–4 months and cell morphology regularly checked. All cells were maintained in Dulbecco's modified Eagle's medium (DMEM) supplemented with 10% FBS at 37°C with 5% CO2. PN1 expression vectors were transfected into cells using FuGENE 6 reagent (Roche). All transfection experiments performed for 24 h unless otherwise noted. Where indicated, inhibitors, recombinant proteins, or blocking antibodies were added to serum free medium during treatments: NF-κB inhibitor Bay11–7082 (BIOMOL Research laboratories, PA), PI3k/AKT inhibitor LY 294002 (Calbiochem), AKT inhibitor MK-2206 (Santa Cruz), recombinant uPA (America Diagnostica), recombinant PN1 (R&D system), uPAR blocking antibody (R&D Systems), and LRP blocking antibody (Progen, Germany). Recombinant human TRAIL was generated as described [45]. uPA activity was measured using a uPA Activity Assay kit (Millipore), per manufacturer's instructions. Measurements were taken at 405 nM on a Tecan m200 plate reader.

### Immunoblotting and immunohistochemistry

Cell pellets were lysed in TNN buffer containing protease inhibitor cocktail (Calbiochem). 50 μg of protein was loaded into 10% Bis-Tris gels (Invitrogen). Blots were probed with the indicated antibodies: anti-PN1, XIAP, DR5 (R&D Systems), beta-actin (Santa Cruz), p-AKT, PARP, all NF-κB molecules (Cell Signalling), and uPA (Abnova).

All immunohistochemistry was performed on tissues harvested from wild type C57/B6 or pn1−/− animals on a B6 background. All harvested tissues were fixed for 24 hours in 4% paraformaldehyde and sliced at 10 μM thickness for staining. Briefly, microscope slide mounted tissues were hydrated and exposed to an antibody at a 1:50 dilution overnight following antigen retrieval (Sodium Citrate, 20 min at 90°C). Pre-diluted Immpact secondary antibody (Vector Labs) was applied to the slides for 30 min and Immpact DAB (Vector Labs) was added to observe positive stain. Samples were evaluated microscopically and photographs were taken on a 20× objective.

### Apoptotic assays

Apoptotic Protein arrays (R&D Systems, ARY009) were used to probe for alterations in protein amounts. PC3 cell lysates were prepared 24 h after transfection with PN1 expression vectors and 300 μg of lysate was diluted in blocking buffer. This solution was incubated with an apoptotic protein array containing 35 pro- or anti-apoptotic proteins overnight at 2–8°C. After 3× washing with PBS, reconstituted Detection Antibody Cocktail was added to the membrane and incubated for 1 hour. The membrane was then subjected to Streptavidin-HRP followed by a chemiluminescent reagent, per manufacturer's instructions.

### XIAP ELISA

All ELISA detection of XIAP protein was performed using the Human Total XIAP DuoSet IC (R&D Systems, DYC822) according to factory instructions. Briefly, 10 μg of cell lysate was combined with a provided XIAP capture antibody fused to the bottom of a 96 well plate overnight. Then a streptavidin-linked detection antibody was applied for 2 h before a substrate was added (20 min) for colorimetric detection at 450 nM using a Tecan m200 plate reader.

### RNA isolation and quantitative RT-PCR

RNA was extracted by Trizol and the RNA concentration was measured by a Nano-Drop 1000 spectrophotometer (Thermo). All qRT-PCR reaction mixtures were prepared using Superscript Platinum III one step kits with incorporated SYBR Green (Invitrogen). One step cDNA production and DNA amplification were performed on a Stratagene MX 3005P thermocycler. All amplified products normalized against GAPDH. Amplified products confirmed by electrophoresis using 1.5% high-resolution agarose gels.

### siRNA

siRNA directed against *pn1*,*upa*, *lrp*, *upar*, and siRNA negative control oligos were from Ambion. 10 nM of each siRNA was transfected into cells using the siPORTNeoFX transfection agent (Ambion). After 48 h, cells were washed and the media replaced with serum free media. Conditioned media was collected 24 h later.

### Matrigel plugs

Briefly, 1 × 10^6^ PC3 cells (suspended in 50 μl of serum free media and 50 μl of Matrigel supplemented with recombinant PN1 as indicated) were injected subcutaneously into the flanks of adult SCID mice as previously described. When distinct individual tumors reached 100 mm3, TRAIL/apo2 was administered at 40 mg/kg, daily by IP, in appropriate treatment groups. Tumor volumes were determined from calliper measurements of the tumor dimensions at the indicated times.

### Tissue array and immunohistochemistry

Human tissue microarrays (PR956b; BioMax) contained samples from 36 cases of prostate cancer with various Gleason scores and 12 examples of normal prostate tissue. TMA staining was assessed using a combination of intensity and percentage of positive stained cells, as previously described [46]. The slides were analysed by two independent pathologists.

### Statistics

All statistical measures were determined using the Prism 5 Graphpad software. Statistical significance was considered to be a *p*-value of < 0.05.

## SUPPLEMENTARY FIGURES


