# Supplementary material for: Protease nexin 1 induces apoptosis of prostate tumor cells through inhibition of X-chromosome-linked inhibitor of apoptosis protein
Source: Oncotarget. 2015 Feb 17;6(6):3784–96. doi: 10.18632/oncotarget.2921 (PMC4414153; doi:10.18632/oncotarget.2921)
Supplement: Supplementary file 1 [file oncotarget-06-3784-s001.pdf]

SUPPLEMENTARY FIGURES

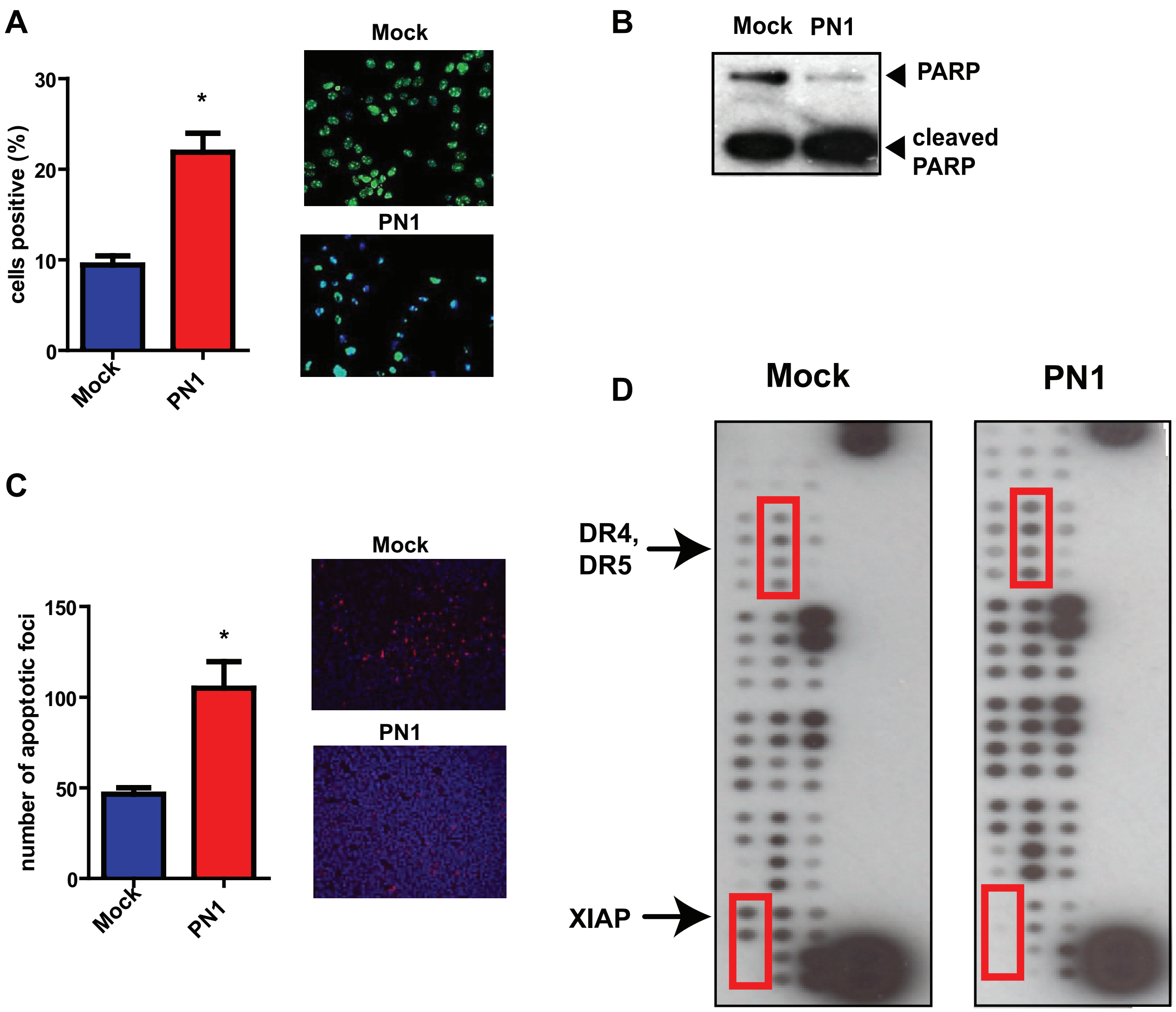

Supplementary Figure 1: PN1 induces apoptosis in prostate metastatic cells. **(A)** PC3 cells (1×10^5) transfected with 2 µg mock or PN1 expressing vector were measured for apoptosis *via* TUNEL. Cells with positive PN1 (green stain) were counted from 9 individual microscopic fields and plotted (*t*-test, $^*P < 0.05$). Blue staining represents nuclei. **(B)** Reduction of full-length PARP to its cleaved form was observed PC3 tranfection. **(C)** Tissues of PC3 xenografts or xenografts pre-treated with PN1 (10 µM) tested for apoptosis *via* TUNEL. Apoptotic foci (red stain) were counted and plotted (*t* -test, $^*P < 0.05$). **(D)** Whole lysates (300 µg) of PC3 cells treated with 2 µg of pcDNA3-PN1 or vehicle only were collected and incubated on an apoptotic array ($N = 3$, one-way ANOVA, $p < 0.05$). Red boxed areas indicate protein level differences in XIAP or death receptors in response to PN1 treatment.

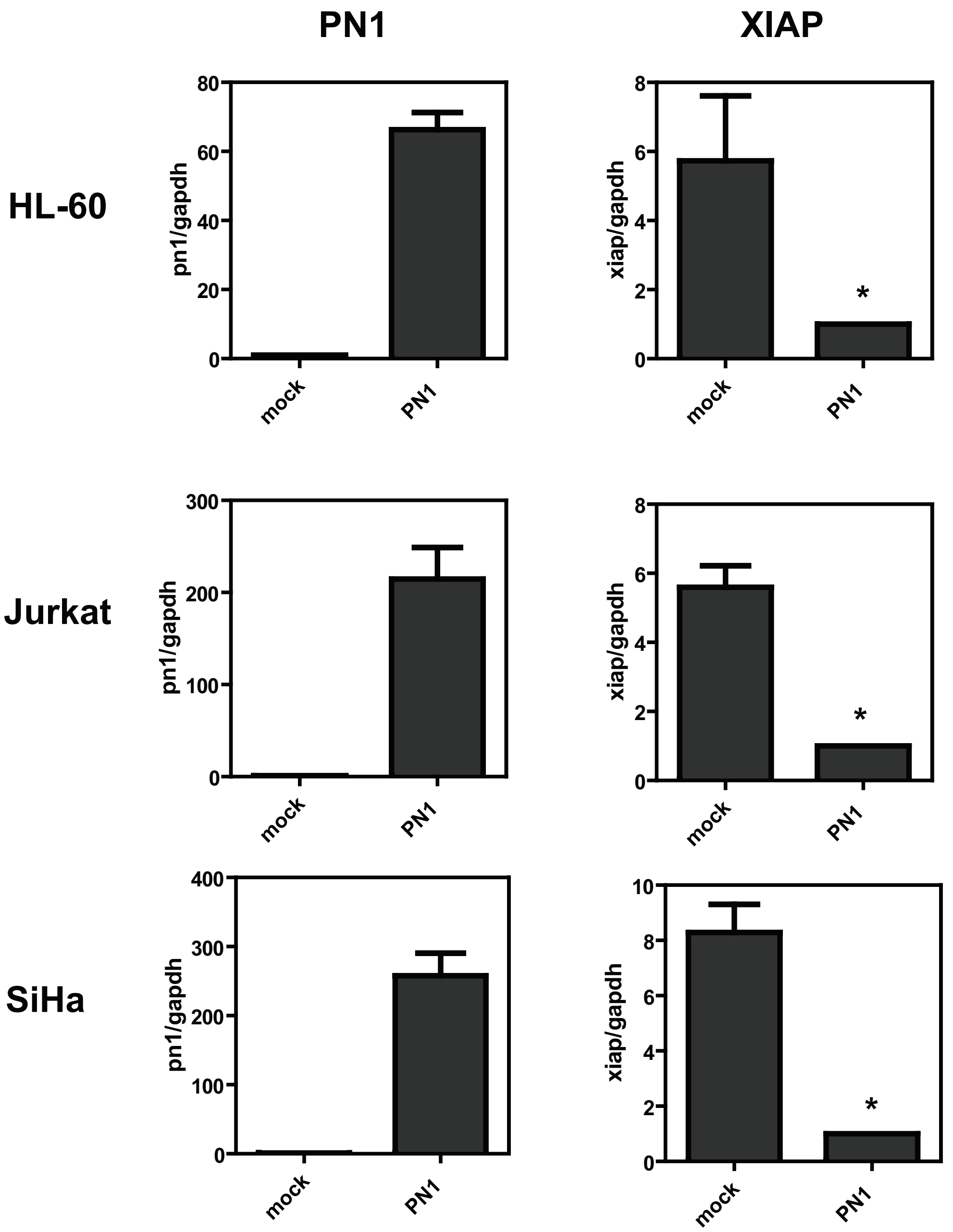

Supplementary Figure 2: XIAP expression is transcriptionally inhibited by PN1 in leukemic and cervical cancer cell lines. Two common human leukaemia cell lines (HL-60 and Jurkat) and a cervical cancer cell line (SIHA) were tested to determine if PN1 expression reduced *xiap* mRNA levels. Transcripts were analysed using qRT-PCR ($N = 3$, t-test, $P < 0.01$).

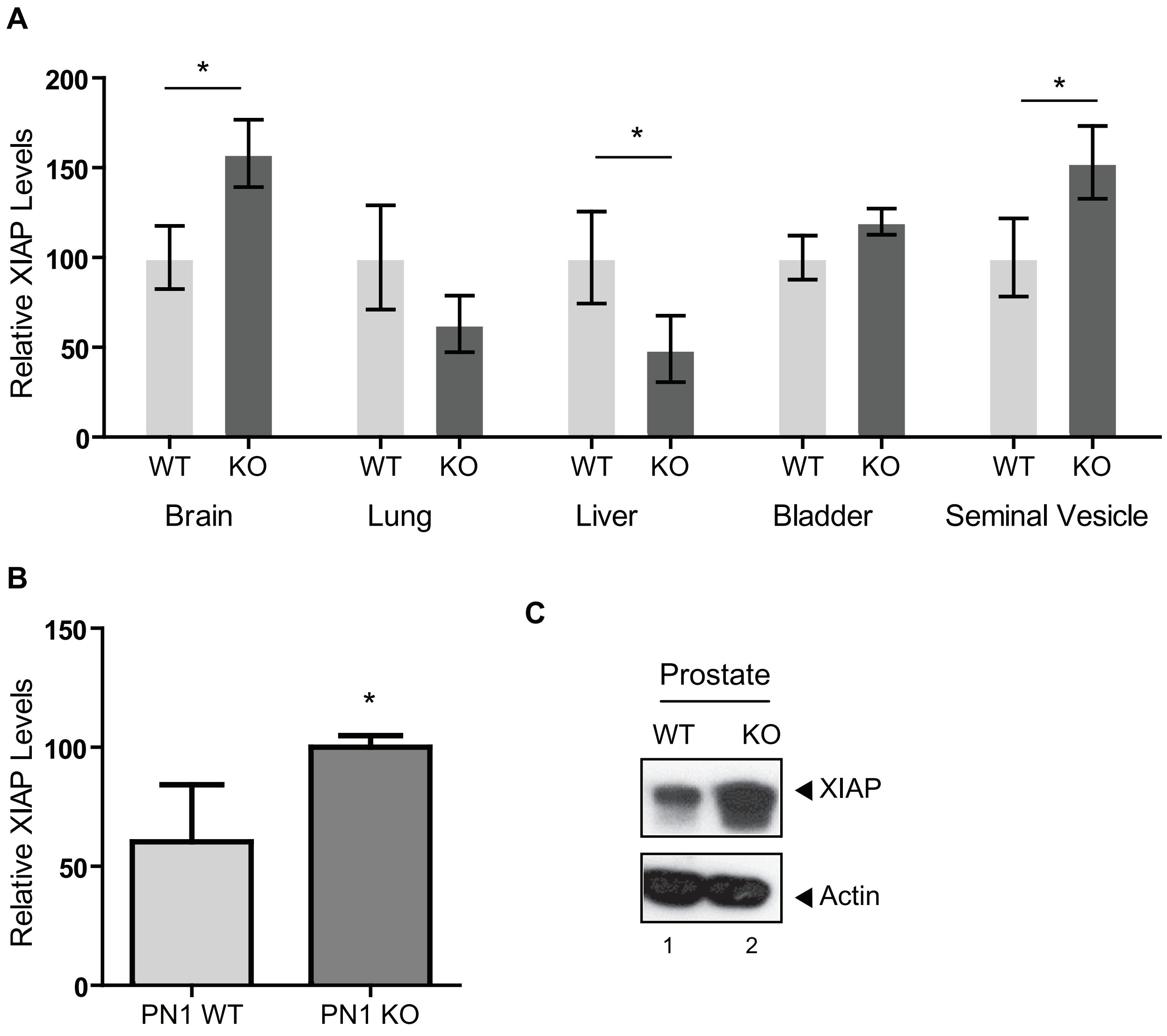

Supplementary Figure 3: XIAP expression is mouse tissues. (A) Lysates of organs from C57B/6 wild type or PN1 KO were tested for differences in XIAP protein levels using a sandwich ELISA ($N = 4$, one-way ANOVA, $*P < 0.01$). **(B)** Lysates of prostates from C57B/6 wild type or PN1 KO were tested for differences in XIAP protein levels using a sandwich ELISA ($N = 4$, one-way ANOVA, $*P < 0.01$). **(C)** Immunoblotting of lysates from wild type or knock-out prostates lysates and probing for XIAP protein ($N = 3$).

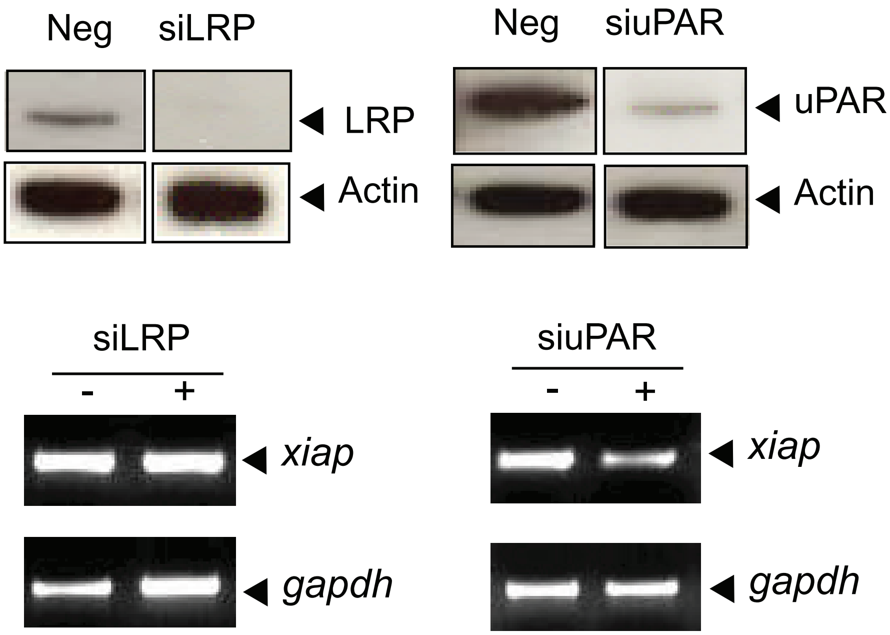

Supplementary Figure 4: uPAR is important for *xiap* transcript expression. Immunoblotting of LRP or uPAR silenced by 10 nM siRNA in PC3 cells (above) and measurements of xiap mRNA following knock-down (below). *gapdh* is used as a template control.
